# Behçet’s disease with latent *Mycobacterium tuberculosis* infection

**DOI:** 10.1515/med-2021-0002

**Published:** 2020-11-21

**Authors:** Yan Shen, Haifen Ma, Dan Luo, Jianfei Cai, Jun Zou, Zhijun Bao, Jianlong Guan

**Affiliations:** Division of Rheumatology, Huadong Hospital, Fudan University, Shanghai 200040, China; Shanghai Key Laboratory of Clinical Geriatric Medicine, Huadong Hospital, Fudan University, Shanghai 200040, China

**Keywords:** latent *Mycobacterium tuberculosis* infection, Behçet’s disease, clinical manifestations

## Abstract

**Objective:**

The aim of this study is to examine the clinical features of patients with Behçet’s disease (BD) in the presence or absence of latent tuberculosis infection (LTBI).

**Methods:**

This was a retrospective study of 232 consecutive patients with active BD hospitalized between October 2012 and June 2017. LTBI was diagnosed based on the positive T-SPOT.TB assay, negative clinical, and imaging examinations.

**Results:**

Among the 232 patients, 68 (29.3%) had LTBI. The frequency, number, and scope of oral ulcers in the BD-LTBI group were significantly more serious than in the non-LTBI group (all *P* < 0.05). Genital ulcers and eye involvement in the LTBI group were significantly higher than in the non-LTBI group (both *P* < 0.01). No active TB was diagnosed during follow-up (median, 27.9 months; range, 3–58 months). The patients with LTBI had signs of liver damage compared with the non-LTBI group. In the LTBI group, the frequency of alanine transaminase >2.0, the upper limit of normal, was higher in the rifampicin subgroup compared with the non-rifampicin subgroup (*P* = 0.033).

**Conclusion:**

Patients with BD and LTBI had worse clinical features than those with BD without LTBI. Rifampicin might be associated with the damage to liver in BD patients combined with latent TB.

## 1 Introduction

Behçet’s disease (BD) is a multisystem inflammatory disorder characterized by recurrent oral and genital ulcers, uveitis, and epididymitis, and with mucocutaneous, articular, gastrointestinal, neurologic, and vascular manifestations [1]. The etiopathological mechanisms of disease development in BD remain elusive, while genome-wide association studies showed human leukocyte antigen and non-human leukocyte antigen associations. Environmental influences and genetic factors may play a role in the etiopathogenetic mechanisms that lead to the development of the disease, indicating the autoimmune and autoinflammatory nature of BD. In fact, the etiology of white plug is not clear, and it is considered to be related to heredity, environment, infection, and immunity at present, and it remains obscure [2]. BD is prevalent in countries along the ancient Silk Road, a route of travel and commerce from the eastern Mediterranean to East Asia [1]. Its incidence is about 14 patients per 100,000 inhabitants in China [3]. Microbial infections such as oral anaerobes [4], herpesviruses, [5] and *Mycobacterium tuberculosis* (MTB) [6,7] are considered to be environmental triggers of BD. *M. tuberculosis* may trigger BD because of molecular mimicking, and *vice versa*, and the dysfunctional immune system in BD may increase the susceptibility to *M. tuberculosis* [6,7].

China is a country with a high prevalence of tuberculosis (TB), accounting for 9% of the global prevalence and with an incidence of 63 per 1,00,000 person-years [8]. Latent TB infection (LTBI) is a state of persistent immune response to stimulation by *M. tuberculosis* antigens, while without clinical evidence of active tuberculosis [9]. Guidelines on the management of LTBI have not yet been developed, and there remain some controversies about the definition [9,10].

Some patients with BD, presumably with LTBI, develop active tuberculosis after treatment with systemic steroids and/or thalidomide [11]. Therefore, there is a need for an effective treatment strategy for those patients, i.e., managing the inflammatory and immune condition, while without compromising the immune system to the point of active TB occurrence. Of note, the use of tumor necrosis factor (TNF)-α blockers has been associated with active TB in patients with BD and LTBI [12,13,14]. There is strong evidence that LTBI treatment can prevent future TB cases in high-risk settings such as recent close contact with an active case [15,16], while there are no data about LTBI management in BD patients.

Immunosuppressants are commonly used in patients with BD, while those drugs can cause liver damage [17,18]. Rifampicin is an often-used antituberculosis drug [19,20], and it can also cause liver damage [21]. Therefore, there is a possibility that patients with BD and LTBI have worst clinical features and outcomes, and there is a possibility that the treatment of both conditions might increase liver damage, while there is no information at present. Hence, the aim of this retrospective study is to examine the clinical features, to detect long-term outcomes of patients with BD with or without LTBI detected using the T-SPOT, and to explore the long-term outcomes of the patients with BD and LTBI using rifampicin.

## 2 Methods

### 2.1 Patients

This was a retrospective study of 232 consecutive BD patients who were hospitalized in the Department of Rheumatology of Huadong Hospital, Fudan University, China, between October 2012 and June 2017.

### 2.2 Inclusion and exclusion criteria

The inclusion criteria were as follows: (1) age ≥18 years, (2) newly diagnosed with BD, and (3) active BD. The exclusion criteria were as follows: (1) patients treated with systemic steroids and/or immunosuppressive agents, (2) patients with active tuberculosis at admission, and (3) patients who with a history of contact with a patient with active tuberculosis. The patients were divided into the LTBI and non-LTBI groups. The study protocol was approved by the institutional review board of the hospital. The need for individual consent was waived by the committee because of the retrospective nature of the study.

All patients were diagnosed according to the international criteria for BD (ICBD) [22]; the 1990 version was used because it was the current version at the beginning of the study period. According to the ICBD, the clinical manifestations of BD include oral aphthosis, genital aphthosis, ocular lesions (anterior uveitis, posterior uveitis, and retinal vasculitis), neurological manifestations, skin lesions (pseudofolliculitis, skin aphthosis, and erythema nodosum), and vascular manifestations (arterial thrombosis, large vein thrombosis, phlebitis, and superficial phlebitis). BD was diagnosed in the presence of oral ulceration plus any two of the following: (1) genital ulceration, (2) typical eye lesions, (3) typical skin lesions, and (4) positive pathergy test [22]. Clinical evidence of active BD was obtained in all included patients; the activity index was assessed using the International Society for BD (ISBD) score (0–20 points), and active BD was diagnosed when ISBD ≥7 points [1]. All patients received the standard treatment tailored to their condition and including corticosteroids, immunosuppressive agents, and biological agents, according to the guidelines [18].

### 2.3 T-SPOT.TB assay

T-SPOT.TB testing was routinely performed at admission. Approximately 8 mL of peripheral blood was collected in lithium heparin anticoagulant tubes from each patient and healthy control for the T-SPOT.TB assay [23]. The control samples were collected from anonymized healthy patients who received health examination during the study period. The T-SPOT.TB tests were carried out according to the manufacturer’s instructions (Oxford Immunotec Ltd, Oxford, UK). The results were presented as the number of spot-forming T cells (SFCs) by the ELISPOT read plate count. Positive and negative results were defined according to the criteria recommended by the manufacturer; positive was defined as (1) ≥6 SFCs/2,50,000 PBMCs to ESAT-6 or CFP-10 antigen after adjustment for the negative control; (2) when the negative control was ≥6 SFCs, number of SFCs ≥2 folds that of the negative control was considered positive (Oxford Immunotec Ltd, Oxford, UK).

### 2.4 LTBI diagnosis and tuberculosis treatment

There is no confirmatory test for the diagnosis of LTBI since there is no unified guideline [9,10]. Compared to TST, T-SPOT.TB has a higher positive and negative predictive value [24]. Here, LTBI was defined by negative chest computed tomography (CT), negative sputum tests, and positive T-SPOT.TB assay.

The patients diagnosed with LTBI received antituberculosis medication: isoniazid for 6 or 9 months; rifampicin alone for 3–4 months; or isoniazid plus rifampicin for 3–4 months [25].

Given the risk of LTB infection increases in patients receiving immunosuppressive therapy or with immune dysfunction [26], it is important to identify LTB infection in BD patients. However, currently, there is no gold standard test for diagnosing LTB infection. Several studies [27,28,29] suggested that IGRAs, including QuantiFERON-TB Gold In-Tube (QFT-GIT), T-SPOT.TB, and TST, are all acceptable for screening of LTB infection. Besides, patients with BD and LTBI showed to have worse clinical features than those with BD without LTBI. No active TB was diagnosed during the follow-up in our study. To our knowledge, the pathogenesis of BD patients without tuberculosis is not related to TB infection, and these drugs are not required for clinical treatment. In recent years, whole-blood interferon-γ release assays (IGRAs), e.g., T-SPOT.TB, were proposed for the diagnosis of LTBI [30]. Accumulating evidences have confirmed that IGRAs specifically screen LTBI, especially in patients undergoing immunosuppressive treatments [31,32,33,34]. Regardless of anti-TNF treatment, long-term screening via the T-SPOT assay may represent a more sensitive approach to identify BD patients with LTBI. Nevertheless, data on the performance of the T-SPOT.TB assay in BD patients are limited.

Currently, T-SPOT is mainly used in clinical diagnosis of LTBI, while due to the high false positive rate of PPD, a limited number of patients undergo this test. There is no combined approach to diagnose LTBI, and it will be the topic of the next research.

### 2.5 Data collection

The following demographic and clinical data at the time of T-SPOT.TB testing were collected: sex, age, duration of BD, genital ulcer incidence, the frequency (numbers/month), number (highest number during an episode), and involvement (buccal mucosa, tongue, lip, palate, posterior pharyngeal wall, and gingiva) of the oral ulcers, nodular erythema, pseudofolliculitis, gastrointestinal ulcers, eye inflammation, arthritis, central nervous system (CNS) involvement, vascular injury, therapeutic regimen, and rifampicin treatment completion (defined as drug compliance of >80%). Laboratory data included counts of red blood cells, leukocytes, and platelets; erythrocyte sedimentation rate; immunoglobulin levels; aspartate transaminase (AST); alanine transaminase (ALT); and estimated glomerular filtration rate (eGFR), and data were tested after the T-SPOT.TB assay and during follow-up.

### 2.6 Follow-up and outcome

All patients were routinely followed up (3-month interval). The patients with LTBI were followed up by chest CT and sputum collection tests every 6 months. The last follow-up was on September 30, 2017.

The long-term outcomes included abnormal liver function (defined as the increased beyond normal levels of AST or ALT) and abnormal renal function (defined as the decreased eGFR beyond normal range between the onset of LTBI treatment and the last follow-up).

### 2.7 Statistical analysis

All statistical analyses were performed using SPSS 19.0 software (IBM, Armonk, NY, USA). Categorical variables were presented as frequencies and analyzed using Fisher’s exact test. Continuous variables were tested for normal distribution using the Kolmogorov–Smirnov test. Continuous data were presented as mean ± standard deviation and analyzed using the Student’s *t*-test or the Mann–Whitney *U* test. Two-sided *P*-values <0.05 were considered statistically significant.

## 3 Results

### 3.1 Characteristics of the patients

The present study included 232 active BD patients (123 men and 109 women). The patients’ mean age was 38.5 ± 12.7 years. Male patients were younger than female patients (37.9 ± 12.2 vs 39.6 ± 11.2 years) (Table 1). Recurrent oral ulcers were the most common manifestation (228/232, 98.3%), followed by genital ulcers (172/232, 74.1%), skin symptoms (132/232, 56.9%), arthritis (69/232, 29.7%), ocular involvement (42/232, 18.2%), gastrointestinal ulcers (38/232, 16.4%), vascular involvement (10/232, 4.3%), CNS involvement (6/232, 2.6%), and epididymitis (5/232, 2.2%). One hundred and eleven patients (47.8%) received glucocorticoids, and 132 (56.9%) patients received immunosuppressants. Biological agents were used in 96 patients (41.4%). The median follow-up of all patients was 27.9 (range, 3–58) months.

**Table 1:** Characteristics of the 232 hospitalized patients with BD

**Characteristics**	
Age (years), mean ± SD	38.5 ± 12.7
Male	37.9 ± 12.2
Female	39.6 ± 11.2
Male, *n* (%)	123 (53.0)
Disease duration (years), mean ± SD	8.4 ± 8.1
**Clinical features, *n* (%)**	
Oral ulcers	228 (98.3)
Genital ulcers	172 (74.1)
Skin involvement	132 (56.9)
Arthritis	69 (29.7)
Ocular involvement	42(18.2)
Gastrointestinal ulcers	38 (16.4)
Vascular involvement	10 (4.3)
CNS involvement	6 (2.6)
Epididymitis	5 (2.2)
**Treatment, *n* (%)**	
Glucocorticoid	111 (47.8)
Immunosuppressive agents	132 (56.9)
Biological agents	64 (27.6)
Follow-up (month), median (range)	27.9 (3–58)

BD, Behçet’s disease; LTBI, latent tuberculosis infection; CNS, central nervous system.

### 3.2 Differences in clinical parameters between the LTBI and non-LTBI groups

There were 68 patients in the LTBI group and 164 in the non-LTBI group. The frequency, number, and scope of oral ulcers in the LTBI group were significantly higher than in the non-LTBI group (*P* = 0.038, *P* < 0.001, and *P* < 0.001, respectively). The incidence of genital ulcers in the LTBI group was significantly higher than in the non-LTBI group (*P* = 0.002). Eye involvement was significantly higher in the LTBI group (*P* < 0.001). The use of TNF-α inhibitors was significantly higher in the non-LTBI group compared with the LTBI group (*P* = 0.001). There were no significant differences between the two groups in terms of sex, age, course of the disease, and other clinical manifestations such as erythema nodosum, pseudofolliculitis, gastrointestinal ulcers, arthritis, vascular involvement, and CNS involvement. There were no significant differences between the two groups in laboratory indexes such as routine blood tests, erythrocyte sedimentation rate, and plasma levels of C-reactive protein (CRP), immunoglobulins, AST, ALT, and eGFR (all *P* > 0.05) (Table 2).

**Table 2:** Demographics, clinical features, and laboratory indexes

Characteristics	LTBI group *n* = 68)	Non-LTBI group (*n* = 164)	*P*
**Before T-SPOT.TB assay**			
Age (years), mean ± SD	38 ± 14	37 ± 14	0.268
Male, *n* (%)	39 (57.4)	84 (51.2)	0.411
BD duration (years), mean ± SD	10 ± 9	8 ± 7	0.087
**Oral ulcers, mean ± SD**			
Frequency (times/month)	2.6 ± 1.7	1.9 ± 1.2	0.038
Number	4.5 ± 2	3 ± 1.5	<0.001
Scope of involvement	3.3 ± 1	2.4 ± 1.1	<0.001
Genital ulcers, *n* (%)	63 (92.6)	109 (66.5)	0.002
Erythema nodosum, *n* (%)	37 (54.4)	74 (45.1)	0.367
Pseudofolliculitis, *n* (%)	24 (35.3)	47 (28.7)	0.495
Gastrointestinal ulcers, *n* (%)	16 (23.5)	22 (13.4)	0.193
Eye involvement, *n* (%)	22 (32.3)	20 (12.2)	0.006
Arthritis, *n* (%)	22 (32.3)	47 (28.7)	0.609
Vascular involvement, *n* (%)	3 (4.4)	7 (4.3)	0.672
CNS involvement, *n* (%)	2 (2.9)	4 (2.4)	0.501
TNF-α inhibitor	8 (11.8)	56 (34.1)	0.001
Leukocytes (×10^9^)	7.5 ± 2.5	6.9 ± 2.5	0.246
Hemoglobin (g/L)	136 ± 15	136 ± 18	0.907
Blood platelets (×10^9^)	239 ± 89	228 ± 81	0.560
Erythrocyte sedimentation rate (mm/h)	27 ± 25	29 ± 26	0.672
C-reactive protein (mg/L)	14.8 ± 11	14.7 ± 12.6	0.990
Immunoglobulin G (g/L)	11.11 ± 3.55	10.86 ± 4.10	0.761
Immunoglobulin A (g/L)	2.42 ± 1.18	2.74 ± 1.19	0.302
Immunoglobulin M (g/L)	1.32 ± 0.61	1.37 ± 1.05	0.839
Immunoglobulin E (IU/mL)	55 ± 48	75 ± 58	0.458
**After T-SPOT.TB assay**			
AST (U/L)	16.15 ± 7.17	16.38 ± 7.77	0.653
ALT (U/L)	17.97 ± 11.52	19.71 ± 13.73	0.483
eGFR (mL/min/1.73 m^2^)	115.53 ± 27.85	123.09 ± 31.25	0.094
Rifampin, *n* (%)	37 (54.4)	0 (0)	<0.001

BD, Behçet’s disease; LTBI, latent tuberculosis infection; CNS, central nervous system; AST, aspartate transaminase; ALT, alanine transaminase; eGFR, estimated glomerular filtration rate.

### 3.3 Outcomes comparison between two groups

Table 3 presents the liver and kidney indexes according to the absence or existence of LTBI. In the LTBI group, more patients had AST ≤ 1.5, the upper limit of normal (ULN) (13.2% vs 3.0%, *P* = 0.006); ALT ≤ 2.0 ULN (10.3% vs 3.0%, *P* = 0.041); and ALT > 2.0 ULN (13.2% vs 2.4%, *P* = 0.002). There were no significant differences in eGFR after BD treatment between the groups.

**Table 3:** Liver and renal function

	LTBI group (*n* = 68)	Non-LTBI group (*n* = 164)	*P*
**AST,** ***n*** **(%)**			
≤1.5 Upper limit of normal	9 (13.2)	5 (3.0)	0.006
≤2.0 Upper limit of normal	2 (2.9)	0 (0)	0.078
>2.0 Upper limit of normal	2 (2.9)	0 (0)	0.078
**ALT,** ***n*** **(%)**			
≤1.5 Upper limit of normal	1 (1.5)	9 (5.5)	0.290
≤2.0 Upper limit of normal	7 (10.3)	5 (3.0)	0.041
>2.0 Upper limit of normal	9 (13.2)	4 (2.4)	**0.002**
**eGFR,** ***n*** **(%)**			
≤60 mL/min	3 (4.4)	3 (1.8)	0.362
≤30 mL/min	0 (0)	0 (0)	>0.99
≤15 mL/min	0 (0)	0 (0)	>0.99

BD, Behçet’s disease; LTBI, latent tuberculosis infection; CNS, central nervous system; AST, aspartate transaminase; ALT, alanine transaminase; eGFR, estimated glomerular filtration rate. *P* < 0.05 was considered statistically significant was shown in bold.

### 3.4 Subgroup analysis

Table 4 presents the liver and kidney indexes according to the absence or existence of rifampicin in the LTBI group. In the rifampicin group, less patients had ALT ≤ 2.0 ULN (2.7 vs 19.4%, *P* = 0.041), and more patients had ALT > 2.0 ULN (21.6 vs 3.2%, *P* = 0.033). There were no significant differences in eGFR after BD treatment between the two groups.

**Table 4:** Subgroup analysis for the presence or absence of rifampicin in the LTBI group

	Rifampicin (*n* = 37)	No-rifampicin (*n* = 31)	*P*
**AST,** ***n*** **(%)**			
≤1.5 Upper limit of normal	4 (10.8)	5 (16.1)	0.722
≤2.0 Upper limit of normal	1 (2.7)	1 (3.2)	1.0
>2.0 Upper limit of normal	2 (5.4)	0 (0)	0.496
**ALT,** ***n*** **(%)**			
≤1.5 Upper limit of normal	1 (2.7)	0 (0)	>0.99
≤2.0 Upper limit of normal	1 (2.7)	6 (19.4)	**0.041**
>2.0 Upper limit of normal	8 (21.6)	1 (3.2)	**0.033**
**eGFR,** ***n*** **(%)**			
≤60 mL/min	2 (5.4)	1 (3.2)	>0.99
≤30 mL/min	0 (0)	0 (0)	>0.99
≤15 mL/min	0 (0)	0 (0)	>0.99
**Therapeutic regimen for BD,** ***n*** **(%)**			
Glucocorticoids	17 (45.9)	11 (35.5)	0.462
Immunosuppressants	37 (100)	31 (100)	–
Biological agents	4 (10.8)	6 (19.4)	0.494
Incomplete rifampicin treatment	5 (13.5)	0 (0)	>0.99

BD, Behçet’s disease; LTBI, latent tuberculosis infection; CNS, central nervous system; AST, aspartate transaminase; ALT, alanine transaminase; eGFR, estimated glomerular filtration rate. *P* < 0.05 was considered statistically significant were shown in bold.

## 4 Discussion

This study aimed to examine the clinical features of patients with BD with or without LTBI detected using the T-SPOT.TB. This study also aimed to examine the long-term outcomes of those patients. The frequency, number, and scope of oral ulcers in the BD-LTBI group were significantly more serious than in the non-LTBI group; similar observations were made regarding genital ulcers, and liver damage was also more important in the BD-LTBI group. No active TB was diagnosed during the follow-up. Of note, rifampicin might be associated with the damage of liver and kidney in patients with BD combined with latent TB.

BD is a multisystemic disorder that may be associated with MTB infection [6,7]. There is strong evidence that LTBI treatment can prevent future TB cases in high-risk settings, such as recent close contact with an active case [15,16,35], while there are no sufficient data about LTBI treatment in BD patients [36]. In addition, considering the inflammatory status of BD [1] and the persistent immune activation status observed in LTBI [9], there is a possibility that patients with BD and LTBI have worst clinical features than those without LTBI, while there is no information at present. The present study suggests that the frequency of LTBI is high among patients with BD in China. The frequency, number, and scope of oral ulcers and the frequency of genital ulcers and eye involvement in the LTBI group were significantly higher than in the non-LTBI group. In addition, no active TB was diagnosed during the follow-up after the patients were treated with prophylactic antibiotics.

Under favorable circumstances, the inactive bacilli will eventually resume metabolic activity and proliferation, leading to the development of active tuberculosis [10]. Such favorable conditions for the development of active TB include immune modulation using systemic steroids, thalidomide [11], and TNF-α blockers [12,13,14]. Therefore, there is a need for an effective treatment strategy for those patients, i.e., managing the inflammatory and immune condition, without compromising the immune system to the point of active TB occurrence.

The lifetime risk of reactivation of TB in patients with LTBI is estimated to be 5–15%, with the majority developing TB disease within the first 5 years after initial infection [37]. In HIV-positive patients, the risk of LTBI progression is significantly higher, with 7–10% per year [9]. There is strong evidence that LTBI treatment can prevent future TB cases in high-risk settings [15,16,35], while the LTBI treatment regimens remain controversial [9,10]. Nevertheless, 3 or 4 months of isoniazid combined with rifampicin could be recommended [35]. In the present study, no active TB was diagnosed during follow-up after the patients were treated with prophylactic antibiotics, while it was a small sample study, and the results need to be confirmed. Indeed, a high incidence of active TB infections are observed during biologics therapy for rheumatic and inflammatory diseases (e.g., rheumatoid arthritis, inflammatory bowel disease, and psoriasis), prompting the need for an appropriate antibioprophylaxis strategy to prevent TB activation in patients with LTBI [38]. Such activations of TB were observed using TNF-α blockers [39] and IL-1 antagonists [40], and International guidelines recommend LTBI screening before initiating any biologic therapy to implement adequate prophylaxis [41].

Th17 and Th1 cells are involved in the severity of the clinical manifestations of BD [42]. In addition, Th1 and Th17 cells are the main effectors during TB [43], and there is a theoretical possibility that LTBI may lead to greater severity of BD symptoms through the Th17 pathway in BD patients. Nevertheless, the exact mechanisms remain to be determined, while we may hypothesize that Th1 and Th17 cells are increased in response to LTBI and that the imbalance between Th1 and Th17 cells and the IL-10 levels results in inflammation and more severe BD. In the present study, IL10, IL-17, Th1, and Th17 were not measured, whereas the results suggest that the worst clinical manifestations seen in BD patients with LTBI are not mediated by the leukocyte levels, C-reactive protein levels, or the levels of immunoglobulins G, A, M, and E. Since the LTBI group showed higher frequency, number, and scope of oral ulcers, higher incidence of genital ulcers, and more frequent eye involvement than the non-LTBI group, immune factors other than the ones available in the present study might be responsible for those worst manifestations. Additional studies are necessary to examine this hypothesis. Notwithstanding, TB itself is able to induce rheumatologic syndromes, and the association of TB with rheumatoid diseases can result in exacerbated symptoms [44].

The combination of rifampicin and isoniazid for 3 months is recommended for potential TB infection [19,20]. The efficacy of this approach has been confirmed to be similar to that of 9-month isoniazid, whereas the compliance was superior [45]. Immunosuppressants commonly used in patients with BD can cause liver damage [17,18] similar to the antituberculosis drug rifampicin [21]. In the present study, the frequency of the elevated ALT was higher in the patients who were taking rifampicin compared with the patients LTBI without rifampicin. Therefore, isoniazid alone might be safer for patients with BD. Considering the long period of treatment, the increased ALT levels can become clinically relevant in some patients.

The present study suffers from some limitations. It was a retrospective study with all the inherent limitations and biases, and the data were limited to those available in the medical charts. In addition, the patients were from a single center, and the sample size was small. Only 10 patients had a second T-SPOT.TB test during follow-up, and we need more patients to evaluate the treatment response. No patient developed clinical symptoms of active tuberculosis, probably because of antibioprophylaxis, while the follow-up was short, and no strong conclusion can be made on this point. Additional studies are necessary to characterize LTBI and active tuberculosis in patients with BD. A large sample size study for exploring the association between rifampicin and liver function is needed.

In conclusion, the present study suggests that patients with BD and LTBI had worse clinical features than those with BD without LTBI. No active TB was diagnosed during the follow-up. Rifampicin might be associated with the damage to liver in BD patients combined with latent TB.
